# *Lactobacillus plantarum* PS128 and Other Probiotics in Children and Adolescents with Autism Spectrum Disorder: A Real-World Experience

**DOI:** 10.3390/nu13062036

**Published:** 2021-06-14

**Authors:** Martina Maria Mensi, Chiara Rogantini, Michele Marchesi, Renato Borgatti, Matteo Chiappedi

**Affiliations:** 1 Child Neuropsychiatry Unit, IRCCS Mondino Foundation, 27100 Pavia, Italy; martina.mensi@mondino.it (M.M.M.); renato.borgatti@mondino.it (R.B.); 2 Department of Brain and Behavioral Sciences, University of Pavia, 27100 Pavia, Italy; chiara.rogantini01@universitadipavia.it (C.R.); michele.marchesi01@universitadipavia.it (M.M.)

**Keywords:** autism spectrum disorder, probiotics, children, adolescents, *Lactobacillus plantarum* PS128

## Abstract

Autism Spectrum Disorder is a neurodevelopmental disorder. Recent data suggest that probiotics can reduce some symptoms of this disorder and *Lactobacillus plantarum* PS128 has been reported to be especially useful. We recruited a sample of 131 autistic children and adolescents (M:F = 122:19; age: 86.1 ± 41.1 months) and evaluated their changes after use of probiotics by mean of CGI. We found some significant improvements with very few side effects; these positive effects were more evident in younger children. Patients taking *Lactobacillus plantarum* PS128 had greater improvements and fewer side effects than those taking other probiotics. Our real-life data are consistent with existing literature showing a specific effect of *Lactobacillus plantarum* PS128 in Autism Spectrum Disorder.

## 1. Introduction

According to the DSM 5 [1], Autism Spectrum Disorder (ASD) is diagnosed in children having persistent deficits in social communication and interaction across multiple contexts, and a restricted and/or repetitive pattern of behaviors, interests or activities. It is a complex disorder, with heterogeneous etiopathogenesis and clinical presentation [2,3]. The etiology, pathogenesis and treatment of choice for ASD are still widely debated topics, as evidenced by the always increasing number of papers published [4]. However, the consistency of the clinical diagnostic criteria is demonstrated by the fact that in the field trials of DSM 5 criteria [5], the diagnosis of ASD had the highest kappa statistic compared to other diagnosis applicable in children (0.69, indicating a very good inter-rater agreement).

In recent years, a growing amount of research has focused the importance of nonpsychiatric factors in this disorder [6]. Given the relatively high prevalence of gastrointestinal disorders in ASD [7], in recent years many scholars have focused their research attention on the gut of these subjects. This line of research led to evidence a higher prevalence of gastrointestinal disorders not specific to ASD, the most common being possibly eosinophilic colitis [8].

Several studies showed a significant alteration of the expected composition of gut microbiota (dysbiosis) in ASD children compared to typically developing subjects [9], although the findings were not consistent with a specific pattern of alteration. The composition of the gut microbiota, or its degree of dysbiosis, has a number of sources: diet, medication and hygiene, as well as numerous maternal factors which include maternal stress, infection and a high-fat diet during pregnancy [10,11]. The frequent use of oral antibiotics in ASD children during the first three years of life is another factor that has also been hypothesized to disturb the natural balance of the gut microbes, while some antibiotics confer benefits [12].

Studying ASD-like animal models, evidence was provided for a significant role of the microbiota for social development [13]. Moreover, it was seen that restoring the normal components of gut microbiota with probiotics could produce significant benefits on behavior [11,14]. Probiotics are living microorganisms, often part of the gut microbiota, whose administration is considered beneficial for human health. They generally belong to Gram-positive taxa (i.e., Lactobacillus and Bifidobacterium genera). Dinan and Cryan [15] have suggested they could be a therapeutic tool useful for altering brain function through their activity in re-establishing the healthy equilibrium of gut microbiota and modulating tissue neurotransmitter levels; this is actually referred to as “psychobiotic” effect [16].

This led to a number of studies concerning the possibility to use the so-called “fecal transplantation”, which is, in fact, a depletion of the microbiota followed by the introduction of a new one, as a treatment for ASD [17]. This approach was, however, criticized due to possible side effects. This, in turn, suggested the opportunity to focus on probiotic supplementation as a much safer way to obtain the psychobiotic effect.

Among other probiotic species, *Lactobacillus plantarum* PS128 has been reported to exert specifical positive effects in children with ASD [18]. *Lactobacillus plantarum* species are rod-shaped, facultative heterofermentative and anaerobic, Gram-positive lactic acid bacteria that can be part of the human gut microbiota. Specifically, *Lactobacillus plantarum* PS128 was isolated from fu-tsai [19]. This traditional fermented food is produced in sealed jars filled with sun-dried mustard plants and salt, which are stored upside down for at least three months. After this fermentation period, lactic acid bacteria can be isolated by homogenization of the fermented mixtures.

Preclinical studies showed that the assumption of *Lactobacillus plantarum* PS128 could increase dopamine and serotonin levels in the prefrontal cortex and in the hippocampus [20,21], and norepinefrine in the prefrontal cortex and in the striatum [22]. Based on these findings, a study was conducted in 71 children and adolescents (age range: 7–15 years) with ASD to test the effects of *Lactobacillus plantarum* PS128 as an adjunctive treatment. Compared to the groups assuming a placebo, after four weeks some significant changes emerged: a reduction of the total score of the Social Responsiveness Scale, a better use of body parts and objects (as measured by the subscale of the Autism Behavior Checklist), a reduction of rule-breaking behaviors (as measured by the specific subscale of the Child Behavior CheckList [23]) and an improvement in attention difficulties and hyperactivity (as measured by the Swanson, Nolan, and Pelham Rating Scale–IV edition [24]).

Although it was not possible to test the change in brain neurotransmission in these patients, it is possible to assume that the changes seen were due to the modification of the levels of monoamines documented in preclinical research [20,21]. This is also in line with the effect on neurodegenerative progression in a murine model of Parkinson Disorder after the administration of *Lactobacillus plantarum* PS128 for four weeks [25], and with the reduction of the severity of tics induced in the experimental murine model of Gilles de la Tourette Syndrome in which *Lactobacillus plantarum* PS128 was administered for two weeks [22]. Noteworthy, in this latter study the effect was related to a change in the gut microbiota induced by the probiotic.

## 2. Materials and Methods

Patients were recruited among those consecutively seen in our third-level center due to spontaneous access or referral from other colleagues for in-depth diagnostic assessment or for a second opinion. Inclusion criteria were having a confirmed diagnosis of ASD and having taken any probiotic at a dose and for a time considered sufficient to allow to detect clinically significant changes (i.e., three months or longer). The only exclusion criterion was parental refusal to provide consent for the study, but all parents accepted to have their children included in the study.

We collected basic data (age, sex, comorbidities), probiotic used and Clinical Global Impression (CGI) scores [26]. The latter is one of the most widely used rapid assessment scales in psychiatry, both in the field of research and clinic. It measures the severity of the disease (CGI-Severity) and the overall change of the patient (CGI Improvement). The severity is measured on a Likert scale ranging from 1 (normal, not at all ill) to 7 (among the most extremely ill patients); the improvement is scored on a Likert scale ranging from 1 (very much improved) to 7 (very much worsened).

Since CGI scores were not normally distributed (Kolmogorov-Smirnov test), nonparametrical statistics were used to assess differences between groups and correlations between variables. Statistical analysis was conducted using IBM SPSS 21 (Armonk, NY, USA). Results were considered statistically significant when the *p* value was <0.05.

The study was approved by the Ethical Committee of the San Matteo Foundation (Pavia) with reference number 20170034734.

## 3. Results

We recruited 131 patients (M:F = 122:19; age: 86.1 ± 41.1 months). Diagnosis of ASD was established from clinical examination and confirmed with the Autism Diagnostic Observation Schedule–2 [27] and the Autism Diagnostic Interview–Revised [28]. All patients received a comprehensive clinical, neurophysiological, neuroradiological and genetic assessment [29,30] from which a potentially causative disorder was diagnosed in few cases (two patients had a diagnosis of cerebral palsy, one patient had Phelan-McDermid syndrome [31] and one had Potocki-Lupski syndrome [32]). Given the very limited number of these subjects with a known potentially causative disorder, no specific analysis was possible in this subgroup (only the patient with Phelan-McDermid syndrome was included in the OP group (see below)).

Most patients had a quite severe form of ASD (CGI-Severity of 5 or higher: 113, 86.3%). Only 52 of them (39.7%) had gastrointestinal problems (most often constipation, with or without behaviors suggesting related pain or discomfort).

105 patients had taken *Lactobacillus plantarum* PS128 (LP group); 26 had been prescribed other probiotics, usually by a pediatrician or by a gastroenterologist seeing them (OP group). Patients in the LP group were prescribed 3 × 10^10^ Colony Forming Units (CFUs) of the probiotic if their weight was less than 30 kg, and 6 × 10^10^ CFUs in they had a higher weight. For the OP group, a patient was considered to be on an adequate dose of probiotics if he/she was taking a dose in the recommended range according to age, weight and specific product.

We defined T0 as the moment when probiotic integration was started; T1 (re-evaluation) was set six months after T0. Compliance was assessed in the interview with the parent/caregiver.

During the study period, all patients continued their treatment as planned by local facilities; treatment was highly heterogeneous (as expected for a real-life “treatment as usual”) but did not change during the study period.

### 3.1. Analysis of the Whole Sample

A clinically significant improvement was seen in the majority of patients: a CGI-Improvement of 3 or lower was assigned to 101 patients (77.1%) and a CGI-Improvement of 1 or 2 to 33 patients (25.2%). Only one patient was judged to be slightly worsened (0.8% of the sample). We observed a correlation between younger age and improvement (Spearman’s Rho = 0.283; *p* = 0.001).

Side effects were reported in six patients: increased irritability in three patients (2.3%) and transitory diarrhea in another three patients (2.3%).

### 3.2. Comparison between Probiotics

Table 1 provides a comparison between subjects in the OP group and those in the LP group.

Patients in the LP group were comparable to those in the OP group from most points of view. However, as depicted in Table 2, they had, on average, somewhat less severe forms of ASD (as measured by CGI-Severity; Mann Whitney: *p* = 0.018). As we could anticipate from the criteria used for their selection, patients in the OP group had significantly more symptoms of gastrointestinal disorders (16, 61.5%, versus 36 of the LP group (34.3%); Mann Whitney: *p* = 0.011).

The difference in severity at T1 increased (Mann Whitney: *p* < 0.001). This was due to a difference in obtained changes: a CGI-I of 3 or lower was reported for 91 patients of the LP group (86.7%) versus 10 in the OP group (38.5%); a CGI of 1 or 2 was obtained by 31 patients in the LP group (29.5%) versus two in the OG group (7.7%) (see Table 3 and Table 4 for details). Most frequently reported positive effects were an increased level of shared attention (54 subjects), a reduction of stereotyped movements (43 subjects), an increase in functional acquisitions in terms of communication skills (32 subjects) and personal autonomies (23 subjects). For some patients improvements in two or more of these areas were reported at the same time.

Side effects were more common in the OP group: in the LP group two patients had an increase in irritability (1.9%) and one had diarrhea (1%), while in the OP group two had diarrhea (7.7%) and one increased irritability (3.8%). This was statistically significant (Mann Whitney: *p* = 0.059).

## 4. Discussion

Our data are in keep with existing literature regarding the utility of probiotics in ASD patients. Moreover, the use of *Lactobacillus plantarum* PS128 proved to exert a more relevant effect. Improvements were significant in terms of global functioning of the patient and were described by caregivers as increased attention, increased communication skills and increased personal autonomies.

The potential effect of probiotics in reducing symptoms in ASD patients has been reported to be independent of gastrointestinal symptoms [14]. In our study, roughly one third of patients in the LP group had gastrointestinal symptoms, but no significant difference was present comparing them to the two thirds without these symptoms. The same analysis was conducted in the OP group, again without any significant difference despite the proportion of patients with or without gastrointestinal symptoms being almost opposite to what was seen in the LP group.

This study also provides evidence of a larger positive effect of *Lactobacillus plantarum* PS128 in children and adolescents with ASD, in line with existing literature on the topic [18]. While the previous paper by Liu and co-workers reported results from a four-week randomized clinical trial, our data provide evidence of a significant effect lasting for six months (measured from the starting of the use of probiotics). The prevalence of detected side effects was low in the global sample, and even lower in the LP group (although this could have been only a consequence of the larger number of subjects included in this group).

A younger age was correlated with a positive effect of probiotics. Observing the plotted graphics, there seemed to be a threshold around age 10, but this was not confirmed by statistical analysis although it seemed substantially in line with previously reported findings [18]. The reason of this correlation can’t be determined by our data. It could be hypothesized either that before that age a higher brain plasticity allows the psychobiotic effect to be stronger and to obtain more significant effects [6], or that after that age the effect of other etiopathogenetic factors tends to become permanent or at least more stable [6,33,34].

This study has some limitations. First, it was conducted without a randomization of participants. Second, the number of subjects in the LP group was significantly higher than those in the OP group. Third, the OP group received a heterogeneous intervention and it was impossible to identify a significant number of subjects being treated according to a single intervention protocol. Fourth, only a clinician-rated scale was used (although the score was decided after a direct observation of the patient and an interview with all adults providing a significant amount of care for the patient). Fifth, the rater was not blinded (i.e., caregivers could tell the rater what the subjects were taking). Sixth, no control group was available for this study. To end with, we did not measure the changes in the gut microbiota and, therefore, the obtained results could not be attributed to a single specific mechanism of action of *Lactobacillus plantarum* PS128.

## 5. Conclusions

This study is in line with existing evidence of a possible beneficial effect of probiotic assumption in children and adolescents, and with the specific utility of *Lactobacillus plantarum* PS128. Its “real life” design, coupled with the longer follow up (six months), confirms the feasibility of this approach, which has a low prevalence of easily manageable side effects, although larger randomized prospective studies are still needed for drawing definite conclusions.

## Figures and Tables

**Table 1:** Subjects’ characteristics at T0.

	OP Group	LP Group
Age in months (M ± SD)	82.4 ± 43	87 ± 40.7
Male:Female	24:2	88:17
Intellectual disability (N (%))	18 (69.2%)	71 (67.6%)
ADHD (N (%))	5 (19.2%)	17 (16.2%)
Anxiety Disorder (N (%))	3 (11.5%)	14 (13.3%)
Drugs taken: risperidone methil-phenydate antiepileptics	2 (7.7%) 1 (3.8%) 1 (3.8%)	9 (8.6%) 3 (2.9%) 0

**Table 2:** CGI-Severity distribution at T0.

CGI-Severity T0	OP Group	LP Group
3	0	2
4	1	15
5	11	55
6	14	33

**Table 3:** CGI-Severity distribution at T1.

CGI-Severity T1	OP Group	LP Group
2	0	1
3	0	15
4	5	52
5	13	31
6	8	6

**Table 4:** CGI-Improvement distribution.

CGI-Improvement	OP Group	LP Group
1	0	3
2	2	28
3	8	60
4	16	13
5	0	1

## Data Availability

Raw data are available from www.zenobo.org and can be accessed via DOI:10.5281/zenodo.4898934.
